# Practical Medicine

**Published:** 1878-08

**Authors:** 


### Practical Medicine.

Sea Sickness. Laederich. (*L'Année Médicale.*)

In answer to an anonymous writer, who speaks ironically of the application of collodion as a remedy against sea sickness, Dr. Laederich, a French army physician, strongly advocates its use, citing well marked cases in defense of his recommendation. He does not pretend to explain the theory of its action, but sees in it a resemblance to the belt which has been used in many cases to advantage, and is frequently recommended by sailors. Dr. L., moreover, looks upon an application of collodion over the epigastrium as a powerful anti-emetic in general, having used it and seen it used in several cases with decided benefit. He imagines that it would thus prove curative as well as preventive of sea sickness.

His method of using it is as follows ; Before embarking the traveler should apply, with a soft brush, three successive layers of collodion containing castor oil, over the region of the epigastrium, being careful to pass beyond its anatomical region. As directions to his patients, he maps out the region thus : A horizontal line directly above the umbilicus ; on the right and left two vertical lines, about five centimeters beyond the nipples ; above, following the curve of the sides, and extending about three or four centimeters above the border. He further recommends those about to take a long voyage to take a supply of collodion with them.

The Nursing of Children.—(*La France Médicale*, p. 357, 1878.)

La Société de Médecine Pratique, through Dr. Brochin, lays down the following rules for the nursing of children :

1. The mother's milk is incomparably the best food for the infant.

2. A wet nurse is preferable to any kind of artificial nursing.

3. Artificial nursing should not be resorted to except in the impossibility of a supply from the mother or a wet nurse.

4. The nursing of a child, unless it is absolutely impossible, should take place in the house where the parents live, whether it is a question of wet nursing or artificial nursing.

5. When labor is completed, a rest of several hours is necessary for the mother. If she is capable of nursing her child, she should give it the breast from five to six hours after the termination of labor.

6. One or two teaspoonfuls of warm water, just a little sweet, should be given the child soon after birth. This practice clears the mouth and throat of mucus and facilitates the discharge of the meconium.

7. It is very desirable from the beginning to regulate the hours for nursing.

8. For the first fortnight it is not desirable to put the child to the breast, or to feed it, oftener than every two hours, and not less often than every three hours.

9. The child should be accustomed to nurse as little as possible during the night. Six or eight hours of uninterrupted sleep being necessary to both mother and child. The physician should decide when it is necessary to infringe on this and the preceding rule.

10. The two breasts should be given to the child alternately. This rule should be observed although he may manifest a decided preference for the one.

11. In case the milk of the mother or nurse is not sufficient, the breast and artificial nursing must be adopted.

12. Cow's milk must be employed fresh without ever being boiled.

13. It is customary for the first week to dilute the milk with three times its volume of water; during the rest of the month with one-half water; from the end of the first month to the end of the sixth month with one-quarter its volume of water; from the end of the sixth month to give it pure. For practical purposes it is sufficient to calculate the quality of the milk by the lactometer.

14. Whatever proportion of milk is used, it is desirable not to sweeten or salt it but very lightly. It should not be prepared but at the time when it is about to be given. It should be heated by placing the bottle in warm water until it has acquired a temperature of from 38° to 40° C.

15. The state of the stools of the child being the best sign of the digestion, it is very important that they should be examined every day. If they are thin, a little more milk should be added; if greenish, grumous, and if they contain undigested milk, the water should be increased.

16. The addition of anything in the way of a narcotic (syrup of poppies, soothing syrup, etc.) to the food is decidedly dangerous, and should be absolutely forbidden without the advice of a physician.

17. Only after the seventh month should the mother's milk or that of the wet nurse be insufficient, is it desirable to supplement the breast with boiled preparations of starch, such as the physician may recommend. This additional food should contain milk as the principal article. It should be always flavored with a little salt and less sugar.

18. Artificial feeding should be subject to the same general principles as the natural nursing.

19. Bottles provided with long tubes which necessitate the child to suck too long, and to cool the milk in its passage, should be discarded and short ones used.

20. Gum drops (suçons) which are frequently placed in the children's mouths to appease their crying, are dangerous, and should be absolutely forbidden.

21. When the child has satisfied its appetite, the bottle and tube should be cleaned immediately, taking its several pieces apart, washing each separately, first in warm, then in cold water.

22. Milk should form the child's food almost exclusively until the first twenty teeth have made their appearance.

23. The weight of the child constitutes one of the surest signs of the adaptability of the food used.

24. Weaning should never take place without the advice of the physician. The usual time for this is between the age of 12 and eighteen months. It is desirable to choose a time when the child is not harrassed by teething, and as far as possible to avoid the period of great heat.

25. The changing of the wet nurse does not endanger in the slightest degree the health of the child.

Action of Urea in the Blood.—MM. V. Feltz and E. Ritter. (*Archives Générales de Médecine*, June, 1878).

The authors give the result of experiments demonstrating that pure urea never determines convulsive accidents. Urea, injected into the blood is eliminated very rapidly by the urine, and when it exists in considerable quantities in the organism, it does not undergo, as is generally believed, a rapid transformation into carbonate of ammonia.

Dogs, which have been injected with urea, after tying the renal vessels to prevent rapid elimination of the poison, did not present convulsive accidents more pronounced than others in which the same vessels had been tied without injecting urea. The convulsions observed after injection of urea were produced by an impure article containing salts of ammonia.

The authors offer the following conclusions: 1st. Pure urea, artificial or natural, injected into the venous system in very large doses, never produces convulsions; it is eliminated very rapidly by the secretions.

2d. There are, in normal blood, no ferments which convert urea into salts of ammonia; the rapidity of elimination cannot be regarded as the cause of this non-conversion, for one may, by suppressing the renal secretion, retard the elimination of urea without hastening the appearance of convulsions.

3d. The ureas, which in large doses caused convulsions, were always impure, containing ammoniacal salts, the presence of which is easily proven by the reagent of Nessler.

Spermatorrhœa and Impotence Caused by Tape Worm.—Houzé. (*Journal de Médicine et Chirurgie Pratique*).

M., 28 years old, has always led a moderate life; no abuse either alcoholic or venereal; never suffered from any affection of the genital organs, health has always been good and functions normal.

In the month of June, 1877, M. perceived that his erections were incomplete; fifteen days afterwards, absolute impotence.

He consulted a physician who prescribed a stimulating diet and some pills. No benefit. Soon after he was afflicted with frequent nocturnal emissions resulting in a very decided lassitude. Defecation caused an expulsion from the meatus of a liquid which was probably prostatic.

His strength diminished rapidly and he became very despondent.

At the expiration of two months he arrived at Paris where a specialist cauterized the prostatic portion of the urethral canal. This was followed by a somewhat violent inflammation. For eight days a mixture of blood and pus was discharged from the urethra. The purulent discharge ceased, but the impotence and the spermatorrhœa persisted.

Houzé saw the patient Nov. 17, 1877. His color was pale; air, sad; eye, restless; speech, spasmodic; respiration, frequent and irregular. Nothing abnormal with the lungs; a slight anæmic murmur was heard at the heart; no pain in the spinal column; catheterism of the canal easy. The finger introduced into the rectum gave no indication of enlargement of the rectum. The inguinal regions revealed nothing abnormal.

Occipital headache; flying pains in the chest; sight diminished and indefinite; moving spots visible; eyelids disturbed with spasmodic twitchings; pupils dilated; hallucinations both of sight and hearing.

He decided to treat him according to symptoms, and prescribed a good nitrogenous diet, iron, cinchona wine, and for the impotence the following pills:

| | |
|---|---|
| Powdered nux vomica,.................................... | 50 |
| Ext. of gentian (sufficient)............................ | |
| Make ten pills. Take two a day. | |

Eight days after, he was rejoiced to inform us that his impotence had disappeared. We continued the same treatment and give in addition a cold douche; allow moderate coition.

The following week, same state and same treatment continued.

Lallemand found in a great number of observations spermatorrhœa to have been the result of intestinal parasites—ascaris vermicularis, and lumbricoides, which disappeared under the influence of anthelmintics.

A decoction of 60 grams of pommegranate bark taken fasting, and three hours later, 30 grams of castor oil brought away five meters and a half of tape worm in several pieces. The following night M. had three polutions.

Since this period the seminal losses have disappeared, the erections are normal; and under constitutional treatment the general vigor appeared forthwith; the sight was restored immediately and the disposition became gay as usual.

The urine examined by the microscope Feb. 5, 1878, did not reveal spermatozoa. In fact, M. at this time was quite cured.

From this it appears that the cause of all these symptoms was the tape worm which had not caused any disturbances of the digestion.

On Some Forms of Chronic Catarrh of the Stomach. Wm. B. Neftel. (*Medical Record*, June 8, 1878.)

The doctor concludes his article with the following propositions:

The local effects of chronic catarrh are—

1. Fermentation in the stomach (butyric acid) with evolution of gases (inflammable) and formation of fatty acids (butyric, lactic, acetic.)

2. Dilatation of the stomach (gastrectasia) and impairment of its peristaltic movements.

3. Stagnation of the food in the stomach and atonic condition of the bowels.

4. Gastric pains and vomiting.

The general effects of gastric catarrh are emaciation and general anæmia from the defective nutrition of the system, especially anæmia of the brain, causing vertigo and other cerebral symptoms.

The diet of those affected with chronic gastric catarrh, though simple and easily digestible, must not be too uniform, and pure water can be used *ad libitum*.

The principal indications in treatment are—

1. To arrest the fermentation by anti-fermentatives, chlorine, fresh prepared according to German pharmacopœia, thymol (very small doses) salicylic acid, hydrochloric acid, etc.

2. To treat the gastrectasia and the atonic condition of the bowels with induced currents. (The "fluctuating induction current," by aid of broad flat electrodes applied to remote portions of the stomach, with the intensity of the current gradually increased from minimum to its maximum, and after several seconds duration again diminished to its minimum. This is repeated fifteen to twenty times in succession, with a pause of several seconds between. The "tetanizing induction current," by applying the broad electrode to one portion of the stomach, the current being of maximum intensity, and suddenly pressing a smaller electrode against the opposite portion for several seconds, then suddenly removing it, and, after a short interval, repeating the process for fifteen or twenty times.)

3. To excite the secretion of a healthy gastric juice by the use of alkaline waters before meals (Vichy, Carlsbad), small doses of rhubarb (gr.i four times a day) ; bitter remedies, etc. (nux vomica, etc.)

4. To improve the digestive properties of the gastric juice by the administration of hydro-chloric acid, not only to dyspeptics but also to anæmic persons generally. A. H. F.

THE CONNECTION BETWEEN PRURITUS VULVÆ AND DIABETES.—Wiltshire. (*Lancet*, Apr. 13.)

The author calls attention to the frequent association of diabetes with pruritus vulvæ, and the desirability of examining the urine of patients suffering from this annoying malady. Aside from the itching, there may be no other symptom of diabetes, and it is not surprising, therefore, that the true condition is overlooked. In his cases, the most successful treatment for the local symptom was a simple borax lotion.

TREATMENT OF OBSTINATE HICCOUGH.—Favier. (*La France Médicale*, May 1st, '78.)

The author reports a very obstinate case of hiccough which persisted for fifty days, resisting all treatment, and which was finally cured by compression of the epigastrium. The instrument employed was Petit's tourniquet, and the hiccough ceased five minutes after its application. At first suspension of compression was followed by return of the complaint, but finally it disappeared altogether.

CASE OF INTESTINAL OBSTRUCTION CURED BY INJECTION OF GAS.—Aribund. (*Lyon Médical, No.* 21, 1878.)

The patient, a dyspeptic, after hepatic colic, had for four days been suffering from symptoms of intestinal obstruction ; alimentary and bilious vomiting, no fæcal matters, intestinal pains, total absence of stools, or of gas by anus, abdomen tympanitic, but slightly painful, slight fever ; his physician had vainly given several purgatives ; baths had been tried and ointments and cataplasms to the abdomen. Croton oil was tried without effect, and the author advised the injection of gas by means of a long canula attached to an artificial seltzer water apparatus. The canula was introduced deeply and at the moment of entrance of the gas the patient felt, as had been expected, a sensation of something tearing inside, accompanied by pain so violent as to cause him to jump out of bed. At the end of some seconds the pain moderated, he lay down, and twenty minutes afterwards, an abundant escape of gas put an end to these alarming symptoms. There remained but a slight amount of gastro-intestinal irritation, the consequence of the purgatives he had taken.

